# Cancer in connective tissue disease

**DOI:** 10.3389/fimmu.2025.1571700

**Published:** 2025-05-09

**Authors:** Antonio Tonutti, Angela Ceribelli, Elisa Gremese, Serena Colafrancesco, Maria De Santis, Carlo Selmi

**Affiliations:** ^1^ Department of Biomedical Sciences, Humanitas University, Milan, Italy; ^2^ Rheumatology and Clinical Immunology, IRCCS Humanitas Research Hospital, Milan, Italy

**Keywords:** malignancy, immunology, autoimmunity, autoantibodies, connective tissue disease (CTD)

## Abstract

The association between cancer and autoimmunity is well-recognized, as represented by the increased incidence of cancer among patients with systemic autoimmune diseases; however, the underlying mechanisms remain only partially understood. On the one hand, malignancy may trigger a breakdown of immune tolerance in predisposed individuals, as autoimmune syndromes often emerge shortly after cancer diagnosis, suggesting that tumor antigens might initiate an autoimmune response. However, by involving persistent responses and the creation of a pro-inflammatory environment, the chronic immune activation characteristic of autoimmunity may promote oncogenesis. This scenario is further complicated by the use of immunosuppressive therapies for autoimmune conditions, which, as seen in transplant immunology, are associated with a higher risk of cancer, although data in rheumatology have not yielded definitive conclusions. Connective tissue diseases include systemic lupus erythematosus, primary Sjögren syndrome, idiopathic inflammatory myopathies, systemic sclerosis, mixed connective tissue disease, and undifferentiated forms. These conditions have been variably associated with an increased risk of cancer, both at the time of disease onset and in patients with long-standing autoimmune conditions, providing a paradigm for investigating this complex interplay. Despite recent progress, many unmet needs remain that warrant further research.

## Why cancer and connective tissue disease

The relationship between malignancy and autoimmunity is well established, as supported by the increased incidence of cancer in patients with autoimmune diseases (1); however, several questions remain unanswered regarding the fundamental mechanisms of this association and their translation into clinical practice. In line with the established pathogenic model of autoimmune diseases, malignancy may trigger the breakdown of tolerance in predisposed individuals (2). This is illustrated by the occurrence of autoimmune syndromes, often with distinctive features, in close temporal proximity to cancer diagnosis (3). On the other hand, autoimmunity may serve as a fertile ground for the development of malignancy, possibly due to persistent immune activation against autoantigens and the setting of a pro-inflammatory milieu, thus acting as a precancerous condition (4). Furthermore, autoimmune diseases are often treated using immunosuppressive therapies. While evidence from transplant immunology indicates that immunosuppression increases the risk of cancer (5), data are inconclusive when it comes to rheumatology and clinical immunology (6).

**Figure 1:**
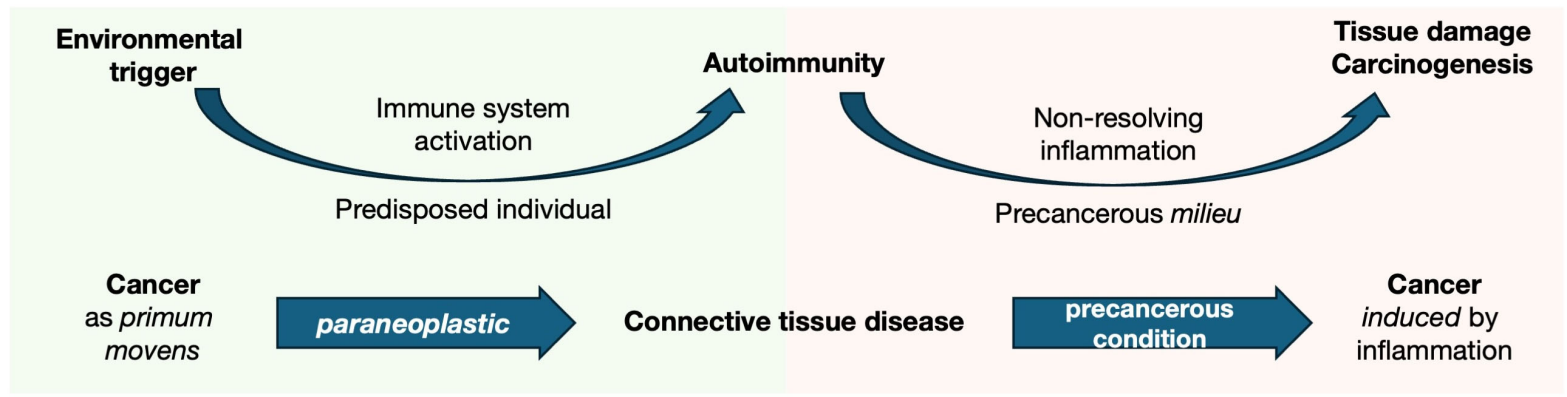
Cancer as both an environmental trigger and pathological consequence of autoimmunity in the paradigm of CTDs. The pathogenesis of autoimmune diseases involves a hypothetical environmental trigger that induces immune system response. In genetically predisposed individuals, this leads to an aberrant immune activation, which becomes dysregulated and persists over time, resulting in chronic inflammation. The chronic inflammatory milieu causes tissue damage due to ongoing inflammation but synchronously provides a precancerous condition (i.e., an environment that predisposes to the development of cancerous lesions). From this perspective, CTD are at a crossroads between cancer and autoimmunity. On the one hand, strong evidence supports the role of cancer as a trigger of autoimmune responses (as seen in cancer-associated myositis and scleroderma). However, the disease itself increases the risk of malignancies, particularly in tissues undergoing chronic inflammatory remodeling (such as the lung in SSc and lymphopoiesis in pSS).

Connective tissue diseases (CTDs) are classic forms of systemic autoimmune disorders, including systemic lupus erythematosus (SLE), primary Sjögren syndrome (pSS), idiopathic inflammatory myopathies (IIM), systemic sclerosis (SSc), mixed connective tissue disease (MCTD), and undifferentiated forms (UCTD) (7–12). These diseases are characterized by unique clinical features and pathogenic mechanisms but also share a female predominance, overlapping clinical manifestations (e.g., arthralgia and arthritis, fatigue, interstitial lung disease, myositis, and Raynaud’s phenomenon) (7–12), and similar immunological pathways (e.g., type I interferon activation, B-cell infiltration, activation, and proliferation) (13, 14). Within this shared framework, an increased risk of malignancy has frequently been reported across CTDs, reflecting the intricate interplay between cancer and autoimmunity (**Figure 1**). We speculate that some entities reflect the causal relationship of autoimmunity as a paraneoplastic phenomenon, as seen in cancer-associated myositis (CAM) or -scleroderma, where the temporal closeness between the two diagnoses is linked to peculiar environmental and pathophysiological changes (15). In other scenarios, subclinical chronic inflammation may constitute a precancerous condition contributing to the development of cancer-associated mutations and malignancy late in disease history (16, 17).

By evaluating the spectrum of CTDs, we present a critical analysis of the relationship between cancer and autoimmunity, with a focus on clinical associations, relevance of serum autoantibodies, impact of disease-specific risk factors, and role of immunosuppressive therapies. Different scenarios will be presented to support the proposed concept that certain CTDs can represent a paraneoplastic phenomenon, whereas the onset of malignancy is observed more frequently in specific longstanding CTD-related contexts. To ensure a consistent approach, similar sections will be summarized for different diseases. However, there are major differences in the available evidence, and considering that our work aims to provide a critical review of the state of the art while identifying clinical and research needs, the content of certain sections will need to be heterogeneous and vary from one condition to another. This is particularly evident in the section on immunological features, which lacks a uniform distribution in myositis and SSc compared to pSS and SLE. **Table 1** summarizes the unmet needs in the management of malignancy in patients with CTDs and outlines a contextual research agenda based on the discussions presented throughout the text.

**Table 1:** The unmet needs and research agenda in cancer management in patients with CTDs.

	IIM	SSc	pSS	SLE
Risk assessment	Can we measure the risk of cancer in new-onset IIM? Can we further stratify the risk in patients with specific phenotypes? (e.g., different autoantibodies associated with DM, ASyS) Can we better stratify patients at high risk according to disease phenotypes and autoantibodies? (e.g., anti-TIF1γ DM who do not develop cancer) Can we assess the risk of IIM in patients newly diagnosed with cancer?	Can we measure the risk of cancer in new-onset SSc? Can we measure the risk of cancer in longstanding SSc? What is the contribution of SSc to this risk? Which sites are the most involved? **In cancer-associated scleroderma**: what are the risk factors beyond anti-POLR3+ dcSSc? **Cancer-associated scleroderma**: which anti-POLR3+ patients will not develop cancer? **Late-onset cancer in SSc**: which patients should be thoroughly and repeatedly screened?	**Lymphoma**: can we measure or score the risk of developing lymphoma in pSS? Can we predict the time-to-lymphoma interval in pSS? Will any novel autoantibody provide more insights in estimating the risk of lymphoma in SSc? **Non-lymphoma**: Is pSS a risk factor for solid neoplasms? Which are the most common neoplasms? What are they associated with?	Which disease categories and phenotypes are at high vs. low risk of cancer? Can we identify any patient cluster? What is the timing of cancer onset in patients with SLE? Which autoantibodies are associated with cancer in patients with SLE, if any? Are overlap diseases (pSS, thyroiditis, autoimmune hepatitis or cholangitis) a concern in patients with SLE? Is elderly-onset SLE a risk factor for cancer?
Screening	How long and how often should patients with IIM be screened for malignancy? Should patients receive long-term screening for specific cancers in case of select internal organ involvement (e.g., ILD)? Does this apply to all IIM patients independently from the risk estimated according to the IMACS guidelines?	**Cancer-associated scleroderma**: How long and often should we screen patients? How should we screen patients? **Longstanding SSc:** When should we start screening patients? How often should we screen them? For which cancers should we screen them? Which diagnostic tests should be used and how should they be used? Which age, if any, should we start or stop searching for cancer in SSc?	**Lymphoma**: How should we screen patients? Which sites should be screened? When should we start screening patients? How often should patients be screened? **Non-lymphoma**: Should patients with pSS be offered a dedicated cancer screening because of specific risk factors?	Should patients with SLE be offered a dedicated cancer screening because of specific risk factors? In patients with overlapping CTDs, should patients follow the same screening procedures according to the overlapping entity?
Treatment	Does immunosuppressive treatment increase the risk of cancer in patients with CTDs? What is the correct management of immunosuppressive therapies in CTD patients with newly diagnosed malignancy? To what extent does disease activity enhance the risk of cancer in patients with CTDs? Which immunosuppressive treatments contribute to reduce vs. increase such risk by controlling disease activity?			

anti-POLR3, anti-RNA polymerase III autoantibodies; ASyS, antisynthetase syndrome; CTDs, connective tissue diseases; DM, dermatomyositis; IIM, idiopathic inflammatory myopathies; ILD, interstitial lung disease; IMACS, International Myositis Assessment and Clinical Studies Group; pSS, primary Sjogren Syndrome; SLE, systemic lupus erythematosus; SSc, systemic sclerosis.

## Methods and search strategy

We conducted a comprehensive critical review by searching PubMed for “idiopathic inflammatory myopathies,” “systemic sclerosis,” “Sjogren Disease,” “systemic lupus erythematosus,” and “cancer.” The search focused on articles published in English from January 2010 to October 2024 and yielded 3,652 results. Papers of key relevance published outside of this period were included if they focused on relevant findings and approaches that could have influenced subsequent publications. Thus, 196 papers were included in the final review. A balanced discussion was provided by including studies that supported or challenged our perspective, ensuring a comprehensive and evidence-based analysis. Multiple reviewers (AT, AC, EG, and SC) independently evaluated the included studies; their interpretation was discussed by the full author panel to minimize bias and reach consensus, and different viewpoints were considered during the synthesis of the results. Owing to the heterogeneity of study designs, patient populations, and outcome measures, which made direct comparisons challenging, a narrative approach was adopted instead of a systematic review. To ensure a broad and speculative perspective on the topic, rigid predefined inclusion and exclusion criteria were not applied. However, studies included were original peer-reviewed research articles, systematic reviews, and meta-analyses. Case reports and small case series were considered only when they provided unique insights into novel clinical associations. Non-peer-reviewed sources and studies were excluded to maintain the robustness of the analysis.

## Cancer and idiopathic inflammatory myopathies: the key role of synchronous malignancy

The heterogenous family of IIM encompasses dermatomyositis (DM), polymyositis (PM), antisynthetase syndrome (ASyS), immune-mediated necrotizing myopathy (IMNM), inclusion body myositis (IBM), juvenile inflammatory myositis, and paraneoplastic myositis or CAM (10, 18, 19). CAM is defined as a malignancy occurs within three years from the onset of myositis in adult patients (20, 21), and the risk of developing CAM varies according to the disease phenotype and the presence of selected myositis-specific autoantibodies (MSA) (22–24). Since the earliest reports dating back to 1916 (25), several studies have confirmed a strong link between cancer and IIM, particularly with DM and in the presence of autoantibodies targeting transcription intermediary factor 1γ (TIF1-γ) and the nuclear matrix protein 2 (NXP2) (26, 27).

### Clinical features of paraneoplastic myositis

DM is the most common IIM clinical phenotype associated with the risk of CAM, presenting as heliotrope rash, Gottron’s sign, or papules (28, 29). Patients with inclusion body myositis and ASyS do not seem to have an increased risk of malignancy (26, 30), even when presenting with signs of DM (30), whereas the risk remains unclear in subjects diagnosed with IMNM (31). In addition to the diagnosis of DM, risk factors for CAM include older age at IIM onset, male sex, smoking history, signs of cutaneous necrosis (32), dysphagia (33), rapidly progressive disease, and elevated inflammatory markers (34–37). Histological features on muscle biopsy, such as minimal lymphocytic infiltration, should also raise suspicion for CAM (38) while interstitial lung disease, arthritis, and Raynaud’s phenomenon correlate with a lower risk of malignancy (34, 36, 37). Different types of malignancies have been reported with CAM, most commonly solid neoplasms, which seem to reflect the incidence observed in the general population. For instance, a large cohort from Northern Europe reported a high risk of ovarian, gastric, colorectal, and pancreatic cancers, and non-Hodgkin’s lymphoma (NHL) (39). In contrast, nasopharyngeal carcinoma was confirmed as the most common neoplasm diagnosed in patients with IIM in the Taiwanese population, followed by lung, breast, and hepatic malignancies (40, 41). Moreover, slight differences in the type of incident neoplasms have been hypothesized by comparing patients with CAM according to the clinical phenotype, i.e., DM vs. PM (39). These differences warrant further investigation across different clinical subsets and ethnicities (**Table 1**).

### Immunological features of paraneoplastic myositis

The immune pathogenesis of CAM involves several complex mechanisms, including the presence of shared antigens between tumor cells and normal tissues, molecular mimicry, and exposure to neo-self-antigens (42). These can be presented to tumor-infiltrating lymphocytes through class I (CD8+ cells) and class II (CD4+ cells) HLA complexes. This process leading to lymphocyte activation may result able to provide cancer elimination; on the other hand, activated lymphocyte may cross react with self-antigens and pathologically infiltrate normal tissues (e.g., skeletal muscle, skin), leading to inflammation and damage (42–44).

Serum autoantibodies, including both myositis-specific (MSA) and myositis-associated (MAA) autoantibodies, are of major use in the diagnosis of IIM and correlate with the development of particular manifestations among different clinical subsets (22). Most importantly, the presence of autoantibodies can further stratify patients with IIM according to cancer risk, as summarized in **Table 2**.

**Table 2:** Myositis-specific and -associated autoantibodies, associated phenotypes and current risk of cancer in IIM patients.

Autoantibody	Target molecule and function	Clinical phenotype	Clinical associations	Cancer risk
anti-TIF1γ/α	Transcription intermediary factor 1γ/α—transcriptional elongation, DNA repair	DM, JDM	DM, no ILD	High
anti-MJ/NXP2	Nuclear matrix protein-2—transcriptional regulation and activation of the tumor suppressor p53	DM, JDM	DM, calcinosis, subcutaneous edema, severe myopathy, dysphagia	High
anti-SAE	Small ubiquitin-like modifier 1 activating enzyme—post-translational modifications	DM	Severe cutaneous disease, dysphagia, systemic symptoms, mild myopathy, mild ILD (50%)	Intermediate
Anti-PUF60 (FIRs)	poly-U-binding factor protein	DM, pSS	Less ILD; in pSS frequently with Ro60, Ro52, La	Intermediate-High (200)
Anti-HMGCR	HMG-CoA reductase—rate-limiting enzyme for cholesterol synthesis	IMNM (statin-induced myopathy)	Necrotizing myopathy	Intermediate
anti-Jo-1	Histidyl-tRNA synthetase	ASyS	Classic ASyS with frequent muscle involvement	Standard
anti-PL-7	Threonyl-tRNA synthetase	ASyS	Severe ILD	Standard
anti-PL-12	Alanyl-tRNA synthetase	ASyS	May present with ILD only	Standard
anti-EJ	Glycyl-tRNA synthetase	ASyS	ASyS, ILD (with anti-Ro52)	Standard
anti-OJ	Isoleucyl-tRNA synthetase	ASyS	ASyS (severe myositis), ILD	Standard
anti-KS	Asparaginyl-tRNA synthetase	ASyS	CADM, ILD, overlap subset with *sicca*	Standard
anti-ZO	Phenylalanyl-tRNA synthetase	ASyS	Classic ASyS, rare (<1% ASyS)	Unknown
anti-YRS (Ha)	Tyrosyl-tRNA synthetase	ASyS	ASyS, rash, arthritis, rare	Unknown
anti-KJ	Translocation factor	ASyS-like	Rare	Unknown
anti-MDA5/IFIH1	Melanoma differentiation-associated gene 5—innate immune responses against viruses	DM, JDM	CADM, severe ILD, peculiar skin involvement (reverse Gottron, vasculitis, ear lesions), mechanic’s hands, MIP-C	Intermediate
anti-TIF1-β	Transcription intermediary factor 1β—regulation of gene expression and chromatin structure	DM	CADM, no ILD	Unknown
anti-Ku	Heterodimer complex of 2 subunits that binds to free DNA termini—DNA repair, transcription regulation	SLE, SSc, MCTD, PM	Raynaud, arthralgia, myopathy, overlap with other connective tissue diseases	Standard
Anti-SRP	Signal recognition particle—co-translational translocation of proteins across the endoplasmic reticulum	IMNM	Necrotizing myositis, myocarditis, low ILD	Standard
anti-PM/Scl	complex of 100 KDa and 75 KDa—processing and degradation of RNAs	PM, DM, SSc, PM/SSc overlap, SLE	ASyS-like (myositis, Raynaud, arthritis, ILD, mechanic’s hands)	Standard
anti-Mi-2	helicase of the nucleosome remodeling deacetylase—transcriptional regulation	DM	Classic DM (no ILD)	Standard
Anti-cN-1A	Cytosolic 5’-Nucleotidase 1A protein – nucleotide hydrolysis	IBM	Bulbar muscle weakness, wrist flexor involvement	Unknown
Anti-FHL1	Four-and-a-Half LIM domain 1—intracellular protein–protein interactions mainly with cytoskeletal proteins	DM, PM	Severe myositis, dysphagia, vasculitis	Unknown
Anti-RuvBL1/2	Ruv BL1/2 double hexame—DNA repair, chromatin remodeling, gene transcription	SSc, PM	Higher age at onset, men, diffuse SSc and myositis overlap, GI dysmotility, myocarditis	Unknown
anti-SMN	Survival of motoneuron complex—transcriptional regulation and small nuclear RNP formation	MCTD, PM	MCTD with clinical features of all components of SLE, SSc and IIM; high prevalence of PAH and ILD	Unknown
anti-Nup	Nucleoporins	Not known	Myositis, ILD, Raynaud	Unknown

Cancer risk is reported as ‘high’ (i.e., increased compared to same-age general population), ‘intermediate’, or ‘standard’ (i.e., not different to same-age general population), according to the recent International Myositis Assessment and Clinical Studies Group (IMACS) guidelines (36). Otherwise, for rarer or novel autoantibodies, an estimate of the risk of cancer is given according to the references in the Table, linked to observational cohort studies, whereas ‘Unknown’ risk is reported if little (e.g., case reports, small case series) or no evidence showing cancer association is available.

ASyS, antisynthetase syndrome (myositis, ILD, polyarthritis, Raynaud’s phenomenon, mechanic’s hands and the presence of an antisynthetase antibody); CADM, clinically amyopathic/hypomyopathic DM; DM, dermatomyositis; GI, gastrointestinal; IBM, inclusion body myositis; ILD, interstitial lung disease; IMNM, immune-necrotizing myopathy; JDM, juvenile dermatomyositis; MCTD, mixed connective tissue disease; MIP-C, MDA5-associated autoimmunity and interstitial pneumonitis contemporaneous to the COVID-19 pandemics; PAH, pulmonary arterial hypertension; PM, polymyositis; pSS, Sjogren syndrome; SLE, systemic lupus erythematosus; SSc, systemic sclerosis.

While malignancies often occur in association with DM, a 2012 meta-analysis including 312 adult patients with DM found that 80% of DM patients with cancer were anti-TIF1-γ-positive, whereas only 10% without cancer had this autoantibody (45). Overall, among patients with DM, the presence of anti-TIF1-γ autoantibodies had a positive predictive value for CAM of 58% and a negative predictive value of 93% (45). These findings were confirmed in another large cohort study, particularly raising concern for breast and ovarian neoplasms (26), and in an up-to-date meta-analysis (34). Moreover, it seems that the risk of cancer significantly increases in patients displaying high anti-TIF1-γ autoantibody titers, specifically in patients with the IgG2 isotype, compared with their respective counterparts (46, 47). TIF1-γ, also known as TRIM33, is an enzyme involved in post-translational peptide modifications, an E3-ubiquitin ligase and being involved in small ubiquitin-like modifications (SUMO). In particular, TIF1-γ has been demonstrated to participate in cell cycle regulation, DNA repair, and the regulation of TGF-β signaling (44). Alterations in the TIF1-γ gene have been described in cancer cells from patients with CAM, possibly representing the neo-self and thus triggering the anti-cancer immune response, which can culminate in autoimmunity to native TIF1-γ antigens (48). As a proof of concept, high expression of TIF1-γ has been observed in the skin and skeletal muscle, which represent the main targets of anti-TIF1-γ DM compared to other tissues (49, 50). Recently, the role of anti-TIF1-γ as a risk factor for synchronous cancer in DM patients has been redefined. Indeed, the coexisting immune response against autoantigens, such as Sp4 and CCAR1, would reduce the risk of cancer, perhaps accounting for a more robust antitumor immunological response (51–53). Further implementation of these observations in clinical practice is required (**Table 1**).

NXP2, also known as MORC3, is a nuclear protein involved in the activation of the tumor suppressor protein p53 (54), a key regulator of cell cycle and senescence. Downregulation of NXP2 has been described in different malignancies, correlating with an enhanced type I IFN signature and, most importantly, with increased expression of the immune checkpoint antigen PD-L1, which is known to suppress T-cell response by binding to the cognate receptor PD-1 (55). Autoantibodies against MJ/NXP2 have been extensively associated with the risk of cancer in IIM patients (27, 56–58), even though some large studies (59) and meta-analyses (60) failed to demonstrate an association with malignancy compared to other patient subsets. The heterogeneity of the results obtained when detecting myositis autoantibodies using different methods (59, 61) suggests that one possible explanation for this discrepancy may be the varying techniques used to identify anti-NXP2 autoantibodies across different studies (58). For instance, in one of the largest studies conducted on anti-NXP2-positive DM, the presence of these autoantibodies was confirmed by immunoprecipitation in only 62% of the patients who tested positive using commercial line blots (59).

Recent studies have reported the risk of malignancy in patients with other rare serum autoantibodies. A higher incidence of cancers was observed with anti-SAE, a hallmark of erythrodermic DM (62–64), with malignancies diagnosed also many years after the onset of myositis in an American cohort (65). SAE1 is a subunit of the E1 complex constituting a SUMO activator protein that plays crucial roles in the activation of type I IFN synthesis but is also involved in tumorigenesis (66). For instance, overexpression of SAE1 has been observed in different types of cancers, correlating with a higher disease burden, metastatic disease, and worse prognosis (67–69). Concerning IMNM, it has been suggested that the risk of developing malignancies increases only in seronegative forms (70, 71), despite some reports suggesting a slightly higher rate in subjects with anti-HMGCR (71–73). Nevertheless, other autoantibodies, namely anti-Ku and anti-Mi-2, have been confirmed not to harbor any increased risk of malignancy in patients with IIM (22, 34, 74). Rare and novel MSA have been identified in short reports of small IIM cohorts, but their association with cancer is still unknown and needs to be studied more extensively in larger cohorts worldwide (**Table 1**). For instance, this is the case with anti-FHL1 (75), anti-RuvBL1/2 (76–78), anti-Nup (79), and anti-SMN (80, 81) autoantibodies, which have been identified in small subsets of IIM patients, as well as in SSc and MCTD.

### Cancer screening in IIM: the IMACS initiative

In 2023, the International Guideline for Idiopathic Inflammatory Myopathy-Associated Cancer Screening was released by the International Myositis Assessment and Clinical Studies Group (IMACS) (36) to provide guidance on the management of patients with suspected CAM. These guidelines enable the stratification of each patient with new-onset IIM into a ‘standard,’ ‘moderate,’ or ‘high’ risk of malignancy, by combining the clinical features, autoantibody status, and demographic factors such as age and sex. For instance, patients should be considered at high risk if they meet at least two of the following criteria: DM phenotype, positivity for anti-TIF1-γ or anti-NXP2, age >40 years at the onset of IIM, persistent high disease activity despite therapy, dysphagia, and cutaneous necrosis. Second, the guidelines outline a ‘basic’ and an ‘enhanced’ screening panel to be performed in a tailored manner in patients with IIM, according to their previously established cancer risk.

Therefore, all patients with IIM should participate in country- or region-specific age- and sex-appropriate cancer screening programs regardless of their individual cancer risk. Additionally, basic or enhanced screening panels should be conducted at the time of diagnosis. The ‘basic screening panel’ should include comprehensive history taking and physical examination, routine laboratory investigations (i.e., complete blood count, liver function tests, acute phase reactants, serum protein electrophoresis, and urinalysis), and chest X-ray. Instead, the ‘enhanced screening panel’ includes total body CT scan, cervical screening, mammography, dosage of the prostate-specific antigen or CA-125 (while other neoplastic markers are not recommended for general screening), pelvic or transvaginal ultrasonography, and search for fecal occult blood. Additional screening with ^18^FDG-PET/CT and upper and lower gastrointestinal endoscopy should be considered in selected patients, based on clinical evaluation.

When evaluated in retrospective cohorts, these recommendations displayed excellent sensitivity in identifying patients with malignancy but with lower specificity. Indeed, most patients with IIM were classified as high or intermediate risk of cancer, with only a minority of subjects being represented in the standard-risk group. The ability of these guidelines to detect patients developing long-term cancers seems comparable to their effectiveness in identifying malignancies occurring close to the onset of IIM (82, 83). Further multicentric, long-term cohort studies are needed to evaluate the application of the IMACS guidelines for cancer screening and their impact on follow-up strategies (**Table 1**). Additionally, there is a recognized need to incorporate emerging evidence on novel risk factors to improve patient stratification (**Table 1**), particularly concerning serum autoantibodies, as outlined in **Table 2**.

## Cancer and Sjogren syndrome: a model of autoimmunity-induced malignancy

PSS is a chronic autoimmune disease characterized by lymphocytic infiltration of exocrine glands, leading to glandular dysfunction and development of systemic manifestations (9). In patients with pSS the overall risk of cancer is higher compared to the general population, with an estimated standardized incidence ratio (SIR) of 2.17 (95% confidence interval—CI 1.57–3.00) (84).

### Clinical features of cancer in pSS

Hematological malignancies are the most frequent life-threatening complication of pSS, with one-third of cancers being B-cell lymphomas (85). Among these, NHL is the most frequently reported, with an SIR of 13.71 (95%CI 8.83–21.29) (84), reflecting a seven to 15 times higher incidence compared with the general population (86). Although autoimmunity-promoting lymphoma is frequently observed in autoimmune diseases, this association is highly expressed in patients with pSS. Mucosal-associated lymphoid tissue (MALT) lymphoma constitutes the majority of pSS-associated NHL cases (up to 65%) and mainly originates from the salivary glands. However, additional mucosal sites can be affected, including the stomach, thyroid gland, and lungs (85). In MALT-NHL, lymphomagenesis represents the last stage of the persistent polyclonal activation of marginal zone B cells. In pSS, this activation can evolve into monoclonality, typically resulting in low- or intermediate-grade lymphomas.

In recent years, efforts have been made to identify clinical features and serological biomarkers that predict the development of MALT lymphoma in patients with pSS. Data from the HarmonicSS cohort identified positive serum rheumatoid factors as the earliest and most persistent independent predictor of lymphoma. Simultaneously, B-cell manifestations (including cryoglobulinemia and glandular, cutaneous, and hematological manifestations) appear to signal a more advanced stage in the lymphomagenesis process (87). Additional biomarkers predictive of a higher risk of NHL development have also been identified, including leukopenia, low complement C4 levels, and presence of anti-La/SSB autoantibodies (88). Major salivary gland enlargement and salivary gland focus score evaluated at the time of diagnosis have also been established as independent risk factors for lymphoma in patients with pSS. In particular, a shorter time interval from pSS to lymphoma has been described with an increasing focus score (89), highlighting the importance of histological evaluation in these patients.

A higher risk of hematologic malignancies, other than lymphoma, has been reported in patients with pSS. In these patients, the detection of monoclonal gammopathy of undetermined significance (MGUS) is common, and as a result, the documented higher prevalence of multiple myeloma is not surprising. The risk of MGUS seems restricted to patients with anti-Ro/SSA and anti-La/SSB autoantibodies (90); however, studies on its evolution to multiple myeloma are limited. Thus, further epidemiological investigations are required to precisely determine the incidence and prevalence of this complication in patients with pSS.

Solid cancers were also more frequently observed in patients with pSS (SIR 1.39). In particular, an association between thyroid and other ENT cancers, nonmelanoma skin cancer, hepatocellular carcinoma, lung cancer, prostate carcinoma, kidney, and urothelial cancers has been reported (84). Among these, thyroid cancer is the most frequently recognized, with a 2.6 SIR reported in a pSS cohort of over 7,000 patients (91). These data were confirmed by Britton Zeron et al., who described thyroid cancer as the most common solid tumor in pSS after hematological neoplasms (SIR 5.05) (92). The explanation for this association remains unclear. However, considering that the risk of developing thyroid cancer is higher in patients with autoimmune thyroiditis (93), and that autoimmune thyroiditis is one of the most frequent comorbidities in pSS (94), it is reasonable to hypothesize that the co-occurring autoimmune disease affecting the thyroid might contribute to the development of this neoplastic manifestation.

Current evidence on the established and putative risk factors for malignancy in patients with pSS is summarized in **Supplementary Table 1**.

### Immunological features of cancer in pSS

MALT lymphoma is thought to result from local antigen-driven B-cell selection within tertiary lymphoid structures (TLS), which are typically referred to as ectopic germinal centers (GCs). It is now recognized that during pSS, ectopic GCs form in the minor salivary and/or parotid glands of approximately 30%–40% of patients (95). Since these structures host crucial phenomena, such as oligoclonal B cell expansion and somatic hypermutation of Ig variable genes (96), ectopic GCs are currently considered the ‘beating heart’ of the autoimmune reaction (97). However, despite these functions, the association between ectopic GC formation and lymphoma development remains unclear. While some studies have indicated that the presence of ectopic GCs in minor salivary gland biopsies is a risk factor for NHL lymphoma development (98, 99), more recent studies have not confirmed their predictive value (100). Nevertheless, the view that ectopic GCs are markers of more active and severe diseases is widely accepted (101). Peripheral biomarkers associated with ectopic GCs formation, such as CXCL13, have been identified (102) and are currently being used in clinical trials to monitor disease progression. Notably, elevated peripheral levels of CXCL13 appear to be associated with an increased risk of NHL, further strengthening the relationship between ectopic GC formation and hematologic malignancy development (103, 104).

### Cancer screening in pSS

Lymphoproliferative disease surveillance remains a challenge in patients with pSS even after stratification according to patient risk. Recent studies have shown that patients without clinical suspicion of lymphoma or increased systemic disease activity are unlikely to benefit from major salivary gland imaging screening for detecting this complication (105). This issue is compounded by evidence of the poor reliability of salivary gland ultrasound protocols and scores in identifying lymphoma in patients with pSS and high clinical suspicion (106). It has been proposed that combining salivary gland ultrasound with histology could improve the detection of patients at the highest risk of lymphoma (106). However, evidence is still lacking regarding optimal screening strategies, imaging modalities, and timing. Efforts should also focus on detecting lymphoproliferative diseases at sites other than the major salivary glands, including both the nodal and extranodal sites. Furthermore, identifying the risk factors and screening protocols for non-lymphoproliferative neoplasms should also constitute a priority in the research agenda (**Table 1**).

## Cancer and systemic sclerosis: a unique scenario for both malignancy-induced autoimmunity and autoimmunity-induced malignancy

Systemic sclerosis (SSc) is associated with an increased risk of malignancy, with cancers being diagnosed at a significantly younger age compared to the general population (17, 107–112), and is a leading cause of death among patients (113–115). Cancer strongly affects the disease course of SSc (110, 116), particularly when diagnosed close to the onset of rheumatological manifestations (117). Breast, lung, and hematologic cancers, including lymphoid and myeloid neoplasms, are most frequently diagnosed in patients with SSc (17, 118–120), but increased rates of melanoma and non-melanoma skin cancers, hepatocellular carcinoma, urothelial (119), and thyroid cancers, particularly in cases of coexistent autoimmune thyroiditis (121), have also been reported.

**Figure 2:**
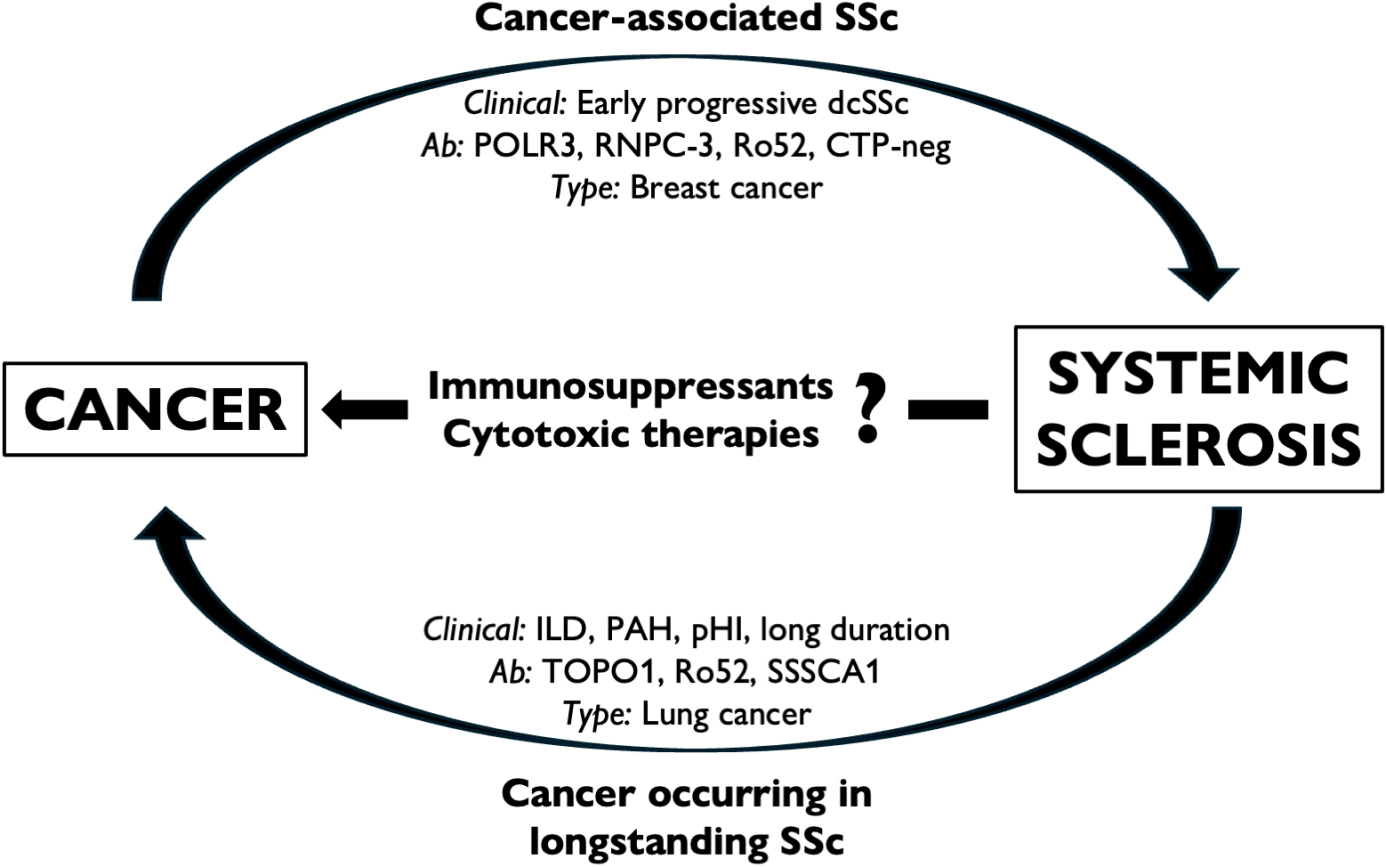
The interplay between cancer and SSc. Some forms of SSc can be regarded as cancer-associated (or paraneoplastic) scleroderma, in which the putative etiological role of malignancy is supposed to trigger the onset of autoimmunity in predisposed individuals (a). Cancer can also occur in longstanding SSc, particularly at specific sites and is associated with the selection of risk factors, phenotypes, and disease complications (b). Immunosuppressive and cytotoxic treatments are commonly adopted to treat SSc-related complications; however, the putative role of such therapies remains elusive (c). CTP-neg, ‘CTP-negative’ patients; dcSSc, diffuse cutaneous SSc; ILD, interstitial lung disease; PAH, pulmonary arterial hypertension; pHI, primary heart involvement.

Risk factors for cancer in patients with SSc include demographic and clinical features, disease duration, selected complications, and the presence (or absence) of particular autoantibodies (120, 122–124). However, a clear profile of the patient with SSc ‘at risk of malignancy’ remains elusive due to the complex interplay between such characteristics and additional risk factors (e.g., family history, exposure to smoking, air pollutants, ionizing radiation, etc.). Compelling evidence suggests that in patients with SSc, some cancers are diagnosed close to the onset of autoimmune manifestations, akin to paraneoplastic phenomena, whereas others exhibit a characteristic delay, often correlating with an increased burden of organ damage (125). These aspects will be discussed in the following sections and summarized in **Figure 2**.

### Clinical features of cancer in SSc

Given the short interval that is seldom observed between the onset of SSc and the diagnosis of cancer, a subset of SSc cases is thought to represent a paraneoplastic syndrome (120, 125, 126), referred to as ‘cancer-associated scleroderma.’ This subset may include patients in whom the antitumor immune response culminates in the onset of autoimmunity (127). From a clinical perspective, early diffuse and rapidly progressive SSc is associated with a high risk of synchronous malignancy (128, 129), particularly in the presence of certain serum autoantibodies.

A second peak of incident malignancies occurs in patients with a long history of SSc and related complications (125), such as pulmonary arterial hypertension and interstitial lung disease (ILD) (119, 124), particularly in cases of progressive fibrosis (120). Chronic inflammation has long been associated with an increased risk of malignancy (4), and what is observed in the SSc scenario could fit within this frame. For instance, this is the case for lung cancer, which arises more frequently in patients with ILD and established disease (123). However, while esophageal involvement is common in SSc, no increased risk of esophageal malignancy has been reported to date. Further research is warranted to test whether the presence of factors considered as ‘protective’ from cancer (i.e., limited cutaneous disease, anticentromere autoantibodies—ACA) (130, 131) is linked to smoldered cancer incidence in this patient subset.

### Immunological features of cancer in SSc

Positivity for anti-RNA polymerase III (POLR3) autoantibodies has traditionally been linked to an increased risk of overall (120, 130, 132, 133) and synchronous cancers (111, 130, 133–135), mostly in patients with diffuse disease (131). Support for the association between the two conditions was elegantly provided by the evidence of alterations in the *POLR3A* locus in samples of synchronous cancers derived from patients with anti-POLR3^+^ SSc, but not in negative cases (15). However, conflicting data on the risk of malignancy with anti-POLR3 autoantibodies have been reported in some cohorts (111, 136, 137). Apart from possibly reflecting genetic or epigenetic differences, such heterogeneity could also indicate the role of multiple autoantibody specificities in modulating the rate of cancers (127, 138). Indeed, similar to what was recently described in DM (51, 52), multiple serum autoantibody specificities likely confer a protective role against malignancy in patients with another autoantibody traditionally linked to an increased risk of cancer. A significant difference in the rate of neoplasms has been observed in anti-POLR3 positive patients with or without concurrent autoantibodies (130, 137). An increased risk of cancer-associated scleroderma has been also reported in patients without anticentromere (ACA), anti-Topoisomerase-I (TOPO1), and anti-POLR3 autoantibodies, the so-called CTP-negative cases (131), as well as in ANA-negative SSc cases (139). Mecoli et al. demonstrated a protective role of anti-Th/To in cancer-associated scleroderma (140). Since the Th/To complex is composed of four molecular subunits (140), it would be useful to investigate correlations between the rate of malignancies based on the presence of single vs. multiple autoantibodies directed towards the different subunits. Similar considerations could be made in patients with anti-POLR3, notably directed to RP155 and/or RP11 subunits of RNA polymerase III (141), and autoantibodies to the PM/Scl complex, which includes a 75 KDa and a 100 KDa subunit and have been associated with malignancy in Spanish patients (120, 142).

Among the rarer autoantibodies, anti-U3-RNP/fibrillarin (138) and anti-RNPC-3, usually associated with limited cutaneous disease but severe organ involvement, have been correlated with cancer-associated scleroderma, along with a worse prognosis, comparable to that observed with anti-POLR3 (143). In particular, a short SSc-cancer interval has been described for anti-RNPC-3 in an American cohort (143), although no association with malignancy was found in another European cohort (144). However, while the first study primarily focused on the characteristics of anti-RNPC-3+ patients and their association with cancer, the European study aimed to characterize the features of patients who tested positive vs. negative for that autoantibody. Moreover, different autoantibody detection methods have been used (143, 144), which could have influenced the results.

Breast cancer is the most frequent malignancy diagnosed as cancer-associated scleroderma, particularly in the presence of anti-POLR3 (136) and diffuse disease (131). Interestingly, breast cancer and SSc share select molecular pathways, including hyperactivation of the mammalian target of rapamycin (mTOR), phosphatidylinositol 3-kinase (PI3K), and transforming growth factor beta (TGF-β) (145). In addition, tumor-infiltrating lymphocytes are more abundant in breast cancers of patients compared than in those without autoimmune disease (145). These observations support the hypothesis of a possible interplay between the anticancer response and the onset of autoimmunity in cancer-associated scleroderma. Further research is required to understand the prognostic role and therapeutic impact of these observations from both the oncological and rheumatological perspectives.

Serum autoantibodies also played a significant role in stratifying patients according to the risk of late-onset malignancy (**Table 1**). Anti-topoisomerase I (TOPO1) positivity is a potential risk factor, particularly for lung cancer. However, it is unclear whether autoantibodies themselves, their association with ILD, or both are putative risk factors for malignancy (110, 146). Late-onset cancer occurs more frequently with the recently described anti-SSSCA1 antibody, an emerging predictor of SSc-related primary heart involvement, which may support the hypothesis of a correlation between long-standing SSc, organ damage, and incident malignancies (147). Anti-SSA/Ro autoantibodies, often detected in patients with SSc and high burden of visceral involvement (148, 149), have been associated to late-onset cancers in a French SSc cohort. A large case–control study attributed this correlation specifically to positivity for the anti-Ro52 subset (130). This result was retrospectively validated by our group in an independent cohort of patients with SSc (137), suggesting a more intricate role of anti-Ro52 positivity. Indeed, cancer-associated scleroderma was more frequently reported when anti-Ro52 was found to be the sole autoantibody, whereas its positivity in combination with other specificities correlated with higher rates of overall cancer throughout the disease history of patients with SSc (137).

**Table 3** summarizes the current evidence on the association between serum autoantibodies and cancer risk in patients with SSc.

**Table 3:** Systemic sclerosis-specific and -associated autoantibodies, clinical associations and current evidence regarding cancer risk.

Autoantibody	Target antigen	Clinical associations	Cancer risk
anti-TOPO1/Scl-70	Topoisomerase I	dcSSc, ILD	Likely increased** (110, 146)
anti-CENP-A/B	Centromere proteins	lcSSc, PAH, DU, calcinosis, gastrointestinal disease	Not increased (130)
anti-POLR3	RNA polymerase III	Rapidly progressive dcSSc, SRC, GAVE	Increased*** (133, 137, 138)
anti-Th/To	RNase P Nucleolar Protein Complex	lcSSc, ILD, PAH	Not increased (140)
anti-NOR90	Nucleolar Organizer Region 90 KDa	lcSSc, mild disease	Not increased (130)
anti-PM/Scl	Nucleolar macro-molecular complex of 75 KDa and 100 KDa	arthritis, myositis, ILD	Likely increased (120)
anti-Ro52	Tripartite motif-containing protein 21	lcSSc, ILD, PAH, overlap pSS	Likely increased^#^ (124, 130, 137)
anti-U3-RNP	Fibrillarin	higher mRSS, myositis	Likely increased^##^ (138)
anti-RNPC-3	RNA Binding Region Containing 3 (U11/U12-RNP)	ILD, gastrointestinal dysmotility	Increased^##^ (143)
anti-SSSCA1	autoantigen p27 (centromere-associated protein)	cardiac involvement*, pSS overlap	Increased^##^ (147)

Due to relatively poor evidence concerning cancer risk, compared to IIM, cancer risk is reported as ‘increased,’ ‘possibly increased,’ or ‘not increased,’ according to relevant literature discussed in the main text. Results are mainly derived from observational cohort or case-control studies. In particular, multicentric cohort studies were available for anti-TOPO1, anti-POLR3, anti-CENP-A/B, anti-Th/To, and anti-PM/Scl autoantibodies.

dcSSc, diffuse cutaneous systemic sclerosis; DU, digital ulcers; GAVE, gastric antral vascular ectasia; ILD, interstitial lung disease; lcSSc, limited cutaneous systemic sclerosis; mRSS, modified Rodnan skin score; PAH, pulmonary arterial hypertension; pSS, Sjogren syndrome; SRC, scleroderma renal crisis.

* Defined as evidence of impaired left ventricle function and/or signs of right failure and/or clinically significant arrhythmia.

** Evidence suggests particularly for long-term incidence of lung cancer.

*** Conflicting evidence pointing towards increased risk only in the absence of multiple autoantibody positivity.

^#^ Evidence suggesting increased risk particularly in patients without multiple autoantibody positivity.

^##^ Evidence from single studies or small case series.

### Cancer screening in SSc

Patients with SSc represent an ideal population for implementing tailored cancer screening strategies because of the potential existence of different risk categories, as recently proposed for IIM (36). Recommendations for cancer screening were proposed by a panel of experts and are specifically meant for patients with new-onset SSc and anti-POLR3 autoantibodies (133). The panel pointed to the need to exclude synchronous malignancy, particularly of the breast, with regular screening suggested thereafter according to age- and sex-related risk factors (133). Despite preliminary evidence demonstrating the predictive role of seriate monitoring of tumor-associated antigen serum levels (150), a panel of experts discouraged their dosage *a priori* in patients with SSc, similar to that in the general population (133). However, the proposed recommendations are only applicable to anti-POLR3 positive patients. Thus, a tailored cancer-screening strategy for SSc remains largely speculative.

Cancer screening should be a priority, and tools to allow patient stratification into different risk clusters are needed. Such clusters may ideally benefit from different screening strategies at different time points during the disease course. As mentioned in the previous sections, the interplay of a wide range of features should be considered to assess the risk of malignancy in patients with SSc, including the disease phenotype, presence and severity of complications, serum autoantibodies, and traditional risk factors, such as tobacco exposure and family history. Finally, it would be interesting to verify whether repeated testing for serum autoantibodies could intercept changes in the autoimmune repertoire, which might help stratify the risk of incident cancer in patients with SSc during the follow-up period (**Table 1**).

### Immunosuppressive treatments and cancer in SSc

Patients with SSc-related organ involvement are treated with immunosuppressive and/or cytotoxic therapies, raising concern for secondary cancers (151, 152) as supported by the observation of urothelial cancers occurring after exposure to cyclophosphamide (119, 120, 153). Mycophenolate mofetil (MMF) is commonly used for the treatment of SSc and is particularly effective in ILD (154, 155). Evidence mostly derived from transplant immunology has not raised major concerns regarding the oncological risk of MMF (155–157), except for the possibly increased rate of non-melanoma skin cancers (158). While no study has specifically evaluated the risk of cancer in patients with SSc treated with MMF, drug safety was suggested in a large cohort of patients treated for fibrotic lung diseases (159), as well as in patients with SSc (138). We hypothesized that the antiproliferative effects of MMF (155) modulate the humoral immune response without affecting cell-mediated immunity (160), thus minimally impairing immune surveillance towards malignancy. Finally, current data are insufficient to establish any association between cancer incidence and more innovative treatments (e.g., rituximab and tocilizumab) in patients with SSc (161) (**Table 1**).

## Cancer and systemic lupus erythematosus: still an unclear scenario

The dual role of immune activation in SLE—driving autoimmunity while potentially influencing tumor suppression or promotion—creates a paradox that is central to understanding the relationship between SLE and cancer. A recent meta-analysis revealed a pronounced increase (2.87-fold; 95%CI 2.49–3.24) in the standardized mortality ratio (SMR) for all-cause mortality among SLE patients compared to the general population (162). Despite the heterogeneity among the included studies, an elevated cancer-related mortality risk (SMR 1.7-fold) was reported in SLE patients (163). The overall cancer risk profile in SLE is shaped by a heterogeneous set of factors, including disease activity and damage, immunosuppressive treatments, genetic predisposition, and environmental exposure (164).

From an epidemiological perspective, SLE displays a unique cancer risk profile. Hematologic malignancies (NHL, Hodgkin lymphoma, leukemia, and myeloma), and lung, cervical, thyroid, gastrointestinal, hepatobiliary, and liver cancers occur more frequently in SLE, which is partly attributed to chronic immune activation and persistent inflammation. Conversely, breast, endometrial, and prostate cancers and melanoma are less common, possibly due to alterations in hormonal pathways and immune surveillance mechanisms (163, 165).

### Clinical features of cancer in SLE

Specific features of SLE, such as hematological and pulmonary manifestations, may contribute to cancer risk, namely NHL and lung cancer. However, despite the well-established association between idiopathic pulmonary fibrosis and lung neoplasms, pulmonary fibrosis is rarely reported in SLE and has not shown statistically significant associations, despite evidence of increasing trends (166). A higher SLICC/ACR Damage Index has emerged as a risk factor for cancer (167, 168); however, the relationship with disease activity risk remains unclear (168) (**Table 1**).

Secondary and overlapping autoimmune diseases, such as Sjogren’s syndrome, autoimmune liver disease, scleroderma, and autoimmune thyroiditis, may contribute to cancer risk in SLE (169) (**Table 1**). For instance, secondary Sjogren’s syndrome increases the risk of NHL (168), although the predominance of the DLBCL subtype raises questions about Sjogren’s status as the primary driver (170). Autoimmune thyroiditis is strongly linked to thyroid cancer in SLE patients, as supported by evidence of thyroid autoimmunity in most cases of thyroid cancer in this population (171).

Childhood-onset SLE (cSLE) is a disease subset that warrants particular attention regarding cancer risk. Lymphomas and solid tumors have been reported at a significant rate, with a median time of 10 years after cSLE diagnosis. Distinct clinical presentations, risk factors, and treatment challenges have been outlined in this population, underscoring the need for heightened vigilance and tailored management strategies for young patients (172).

Finally, patients with SLE may be more susceptible to oncogenic viruses such as Epstein–Barr virus (EBV) (169), human papillomavirus (HPV), and hepatitis B virus (HBV). Impaired immune surveillance could lead to higher rates of viral persistence and reactivation, contributing to the development of lymphomas (173), cervical dysplasia and cancer (174), and hepatocellular carcinoma. By weakening the antiviral defenses, immunosuppressive therapies may further increase this risk.

Current evidence on the established and putative risk factors for malignancy in patients with SLE is summarized in **Supplementary Table 2**.

### Immunological features of cancer in SLE

Chronic inflammation plays a key role in fostering a pro-oncogenic microenvironment via DNA damage, oxidative stress, and cytokine-mediated pathways (175, 176). For instance, the increased risk of lymphoma may be driven by cytokines upregulated in SLE, such as BAFF, APRIL, IL-6, and IL-10, which promote B-cell survival, proliferation, and inflammation (177). These factors are linked to non-germinal center B-cell-like DLBCL, the predominant lymphoma subtype in SLE (169, 178).

SLE-associated autoantibodies, a hallmark of the disease, are hypothesized to promote tumor development by entering cells and causing DNA damage (179). Notably, an anti-DNA autoantibody named 3E10 has been shown to enter cell nuclei, bind to DNA, and impair key DNA repair pathways, thereby contributing to genomic instability. By increasing susceptibility to DNA damage, 3E10 provides a compelling link between SLE autoimmunity and malignancy (180).

Moreover, specific genetic variants (e.g., SNPs in CD40 and HLA alleles) have been associated with both SLE and malignancy, particularly DLBCL and lung cancer (181), although some findings suggest pleiotropy or linkage disequilibrium rather than direct biological causation (182). Emerging research has also identified epigenetic mechanisms, particularly microRNA dysregulation, implicated in both SLE pathogenesis and hematologic cancers, highlighting the potential role of shared post-transcriptional regulatory pathways in the concurrent development of autoimmunity and malignancy (183).

SLE might also confer protection against hormone-sensitive cancers, possibly because of lower exposure to estrogens and androgens. Indeed, women with SLE often experience earlier menopause (184) and are less frequently prescribed estrogen-containing medications (185), whereas men with SLE have lower androgen levels (186). Moreover, certain autoimmune mechanisms may yield protective effects, as in the case of 5C6 anti-DNA autoantibodies that selectively target tumor cells with defects in DNA repair processes (e.g., BRCA2-deficient cancer cells) (187). While the rates of hormone-susceptible breast cancers are similar among SLE patients and the general population, patients with SLE experience a significantly lower incidence of triple-negative cancers, which are mostly characterized by genetic mutations in DNA repair pathways (188).

### Cancer screening in SLE

Established recommendations for cancer screening in patients with SLE are unavailable. Thus, these procedures largely rely on expert opinions, substantially overlapping with what is recommended in the general population (189). In particular, cervical screening and/or HPV vaccinations, periodic mammograms, and fecal occult blood testing are advised for all patients according to age- and sex-specific local guidelines (189). Moreover, clinical screening through regular lymph node examination and routine chemistry is recommended for hematological malignancies, while thyroid enzymes, autoantibodies, and ultrasound should be performed because of the risk of thyroid neoplasms (189). Apart from pursuing smoking cessation, lung cancer screening with annual chest CT scans is recommended only in patients with a high-risk profile (i.e., aged 50 years–75 years and with a history of smoking) (189), while hepatobiliary screening is not recommended unless in cases of positive HBV or HCV serologies (189), and urinary cytology is recommended periodically in patients who have undergone cyclophosphamide.

However, a large cohort study demonstrated that adherence to cancer screening is an issue in patients with SLE, with at least 25% of patients not being regularly screened, particularly in cases of established and longstanding disease (190). This seems particularly crucial regarding cervical cancer screening, since patients with SLE are at higher risk of abnormal test results compared with controls (191).

### Immunosuppressive treatments and cancer in SLE

Immunosuppressive treatments can influence the risk of cancer in SLE (192) because their long-term use may impair immune surveillance (193). Prolonged and cumulative high-dose cyclophosphamide has been strongly linked to an elevated risk of bladder cancer (with oral cyclophosphamide) and hematological malignancies (189). Similarly, azathioprine has been associated with a risk of hematologic malignancies (164), highlighting the need for careful monitoring and optimal dosing. Moreover, the use of immunosuppressive therapies is associated with a higher risk of cervical neoplasia than antimalarials (194), underscoring the importance of regular screening in these patients.

Calcineurin inhibitors have been associated with an increased incidence of cancers in solid organ transplant recipients (195), with previous studies suggesting their role in impairing DNA repair, promoting angiogenesis, and facilitating tumor invasion (196). However, a recent large cohort study of SLE patients with consistent follow-up found no significant difference in cancer risk between those using calcineurin inhibitors and those who did not, even after adjusting for potential confounders (197). Biologics that target B-cell pathways, such as rituximab and belimumab, are generally considered safe; however, their effects on cancer remain the subject of ongoing investigation. Finally, owing to the close association between drug exposure and disease activity, many studies face challenges in distinguishing the individual contributions of these factors to cancer risk (**Table 1**).

Compared to immunosuppressants, hydroxychloroquine, which is universally prescribed for SLE, has been associated with a decreased cancer risk (198), particularly for breast and non-melanoma skin cancer (193), possibly because of its anti-proliferative and anti-angiogenic activity.

## Limitations and concluding remarks

While this study aimed to provide insight into the dual-faceted clinical relationship between cancer and CTDs (i.e., cancer-associated CTDs vs. cancer occurring subsequently or within the context of CTDs), we acknowledge certain limitations. Although our literature review was comprehensive and sought to analyze evidence that supports and challenges our hypotheses, we did not follow a systematic review approach, which would be necessary to address more specific research questions based on the current evidence. A consistent approach was attempted across diseases, but the major differences in evidence availability led to some degree of heterogeneity, particularly in the immunological feature sections related to myositis and SSc *versus* pSS and SLE. Publication bias should also be considered, particularly regarding data on rare and emerging autoantibody specificities, along with the relatively greater abundance of studies on certain diseases, primarily IIM and SSc, compared to pSS and SLE. There are also biases in the races and ethnicities that have been studied in different diseases, which should be addressed in future investigations. The heterogeneity of analytical methods for autoantibody detection (e.g., immunoprecipitation, line blot, and ELISA) should also be considered when comparing different studies, as the sensitivity and specificity vary depending on the techniques used and the target autoantigen (199). Moreover, our objective was to highlight unmet needs and identify avenues for future research in autoimmunity and rheumatology, with potentially significant implications from the clinical, pathophysiological, and therapeutic perspectives.

Patients with CTDs exhibit distinct cancer risk profiles, which are influenced by the etiological role of malignancy in certain contexts and the precancerous environment created by chronic inflammation and autoimmune activation. Similarities in immune pathogenesis are thought to occur among patients with paraneoplastic forms of CTDs, as seen when comparing findings from anti-TIF1-γ+ DM and anti-POLR3+ SSc, in which the complex interplay between cancer-related mutations and aberrant tumor immune editing is thought to culminate in the activation of self-reactive lymphocytes, ultimately leading to tissue damage and CTD onset. On the other hand, chronic immune activation reflecting specific pathogenic clues can be considered a potentially premalignant condition, as suggested by the evidence of an increased risk of lung cancer in patients with longstanding SSc-ILD. From this point of view, the example provided by pSS is paradigmatic, since the disease itself is responsible for the generation of autoreactive lymphocyte clones with lymphoma-prone behavior, ultimately culminating in MALT-NHL onset. Most importantly, a correlation between disease activity and lymphoma risk has been clearly demonstrated in pSS. The role of immunosuppressive therapies in cancer risk in these patients remains unclear. Therefore, further research is needed to unravel the complex interplay between CTDs and malignancy, which requires a multidisciplinary approach that integrates clinical and pathophysiological aspects (**Table 1**). Addressing this challenge is essential to improve cancer screening, prevention, and treatment strategies in this patient population.
