# Supplementary material for: Cancer in connective tissue disease
Source: Front Immunol. 2025 May 9;16:1571700. doi: 10.3389/fimmu.2025.1571700 (PMC12098390; doi:10.3389/fimmu.2025.1571700)
Supplement: Supplementary file 1 [file Table1.docx]

**Supplementary Table 1.** Disease-specific candidate risk factors for lymphoproliferative and most common solid malignancies in patients with pSS.

| **Type of malignancy** | **Candidate predictors** | | | **Cancer risk** | **LoE** |
| --- | --- | --- | --- | --- | --- |
|  | **Serum autoantibodies** | **Target autoantigens** | **Clinical features** |  |  |
| NHL | RF (cryoglobulinemia)  anti-La/SSB | Fc domain of Ig  47kDa phosphoprotein interacting with small RNAs | Low C4  Leukopenia Lymphopenia  Exocrine manifestations  Purpura | Increased  Increased | Case-control (85) and retrospective cohort (86)  Case-control (85) and retrospective cohort (86) |
| MGUS | anti-Ro/SSA  anti-La/SSB | Ro52/TRIM21 is an E3-ubiquitine ligase and cytosolic Fc receptor; Ro60/TROVE2 is a RNA-binding protein acting as a checkpoint for misfolded RNA  47kDa phosphoprotein interacting with small RNAs | N/A | Increased  Increased | Retrospective cohort (88)  Retrospective cohort (88) |
| Thyroid cancer | N/A | N/A | Overlap autoimmune thyroiditis | Possibly increased | Retrospective cohort (89) |

*Abbreviations* – C4: complement fraction 4; Fc: constant portion of antibodies; Ig: immunoglobulin; LoE: level of evidence; MGUS: monoclonal gammopathy of uncertain significance; N/A: not applicable; NHL: non-Hodgkin lymphoma; RF: rheumatoid factors.

*Notes* – Compared to Table 2 and Table 3, structural differences reflect the limited availability of robust evidence concerning the role of serum autoantibodies in assessing the risk of cancer in pSS, apart from MALT-NHL.

**Supplementary Table 2**. Disease-specific candidate risk factors for the common malignancies in patients with SLE.

| **Type of malignancy** | **Candidate predictors** | **Cancer risk** | **LoE** |
| --- | --- | --- | --- |
| General risk factors | Childhood-onset SLE  Higher SLICC/ACR Damage Index | Increased  Increased | Retrospective cohort (170)  Case-control (166) and retrospective cohort (165) |
| Hematologic  > NHL | Secondary or overlap Sjogren Syndrome  EBV infection  Hematological manifestations | Increased  Increased  Increased | Case-control (166) and retrospective cohort (165) [for all predictors] |
| Lung | Lupus pneumonitis | Possibly increased | Retrospective cohort (164) |
| Thyroid | Autoimmune thyroiditis | Increased | Expert opinion (168) |
| Cervical | HPV infection | Increased | Prospective cohort (172), retrospective cohort (189) |
| Hepatobiliary | HBV infection  Liver involvement/Overlap autoimmune hepatitis | Increased  Unknown | Expert opinion (187)  Authors’ personal opinion |
| Colon, stomach | N/A | Unknown | N/A |
| Breast – hormone sensitive | N/A | Possibly reduced | Authors’ personal opinion |
| Breast – triple negative | anti-DNA autoantibodies, subtype 5C6 | Possibly reduced | Author’s personal opinion |

*Abbreviations* – EBV: Epstein-Barr virus; HBV: hepatitis B virus; HPV: human papillomavirus; LoE: level of evidence; N/A: not applicable; NHL: non-Hodgkin lymphoma; SLICC/ACR: Systemic Lupus Collaborating Clinics/American College of Rheumatology.

*Notes* – Compared to Table 2 and Table 3, structural differences reflect the very limited availability of robust evidence concerning the role of serum autoantibodies in assessing the risk of cancer in SLE.

In the absence of available evidence, ‘expert opinion’ is here recognized according to the presence of existent expert consensus (referenced in parentheses) or the Authors’ expert opinion, based on preliminary findings, as exposed in the manuscript.
